# Comparison of risk assessment strategies incorporating coronary artery calcium score with estimation of pretest probability to defer cardiovascular testing in patients with stable chest pain

**DOI:** 10.1186/s12872-023-03076-3

**Published:** 2023-01-28

**Authors:** Jia Meng, Hantao Jiang, Kai Ren, Jia Zhou

**Affiliations:** 1 Department of Kidney Disease and Blood Purification, The Second Hospital of Tianjin Medical University, Tianjin, China; 2 Department of Cardiology, Tianjin Chest Hospital, Tianjin, China

**Keywords:** Coronary computed tomographic angiography, Pretest probability, Obstructive coronary artery disease, Risk group, Major adverse cardiovascular events

## Abstract

**Background:**

The risk assessment of patients with stable chest pain (SCP) to defer further cardiovascular testing is crucial, but the most appropriate risk assessment strategy remains unknown. We aimed to compare current strategies to identify low risk SCP patients.

**Methods:**

5289 symptomatic patients who had undergone coronary artery calcium score (CACS) and coronary computed tomographic angiography scan were identified and followed. Pretest probability (PTP) of obstructive coronary artery disease (CAD) for every patient was estimated according to European Society of Cardiology (ESC)-PTP model and CACS-weighted clinical likelihood (CACS-CL) model, respectively. Based on the 2019 ESC guideline-determined risk assessment strategy (ESC strategy) and CACS-CL model-based risk assessment strategy (CACS-CL strategy), all patients were divided into low and high risk group, respectively. Area under receiver operating characteristic curve (AUC), integrated discrimination improvement (IDI) and net reclassification improvement (NRI) was used.

**Results:**

CACS-CL model provided more robust estimation of PTP than ESC-PTP model did, with a larger AUC (0.838 versus 0.735, *p* < 0.0001), positive IDI (9%, *p* < 0.0001) and less discrepancy between observed and predicted probabilities. As a result, compared to ESC strategy which only applied CACS-CL model to patients with borderline ESC-PTP, CACS-CL strategy incorporating CACS with estimation of PTP to entire SCP patients indicated a positive NRI (19%, *p* < 0.0001) and a stronger association to major adverse cardiovascular events, with hazard ratios: 3.97 (95% confidence intervals: 2.75–5.72) versus 5.11 (95% confidence intervals: 3.40–7.69).

**Conclusion:**

The additional use of CACS for all SCP patients in CACS-CL strategy improved the risk assessment of SCP patients to identify individuals at low risk.

## Background

In daily clinical practice, risk assessment of stable chest pain (SCP) to facilitate decision-making, such as deferral of cardiovascular testing, is important but still a challenge [1, 2]. Previous guidelines [3] recommended traditional pretest probability (PTP) models based on invasive coronary angiography (ICA), which has been demonstrated to overestimate the actual prevalence of coronary artery disease (CAD) [4–8]. The 2019 European Society of Cardiology (ESC) guideline [9] recommended ESC-PTP model [1], which was derived from most contemporary SCP cohorts [10, 11] and revealed robust predictive performance in external validation studies [12–15]. For patients with ESC-PTP < 5%, no further cardiovascular testing is needed and for patients with ESC-PTP > 15%, cardiovascular testing should be referred [16]. For patients with ESC-PTP between 5 and 15%, the additional analysis of other risk factors such as coronary artery calcium score (CACS) can improve the estimation of PTP [16]. However, it remains debatable whether these parameters should be limited to only patients with borderline ESC-PTP.

Recently, using data from large cohorts of symptomatic patients who underwent coronary computed tomography angiography (CCTA), a CACS-based model was developed for the estimation of PTP and the external validation and comparison conducted in the original study overwhelmingly supported the CACS-weighted clinical likelihood (CACS-CL) model [14]. According to data from the original study, low CACS-CL (< 15%) was associated with a low prevalence of obstructive CAD [14]. Although CACS-CL model has been demonstrated to provide robust prediction of obstructive CAD in patients with borderline ESC-PTP, no research has systematically compared CACS-CL model alone-based risk assessment strategy (CACS-CL strategy) and 2019 ESC guideline-determined risk assessment strategy (ESC strategy) sequentially combining two models to identify low risk patients. Moreover, unlike ESC strategy only applying CACS-based estimation of PTP to patients with borderline ESC-PTP, CACS-CL strategy may obviously increase the use of CACS.

Consequently, we aimed to validate and compare ESC-PTP and CACS-CL model in a CCTA-based SCP cohort. Moreover, we aimed to compare ESC strategy and CACS-CL strategy which both incorporated CACS with estimation of PTP and investigate whether the additional use of CACS for all SCP patients in CACS-CL strategy would be efficient in identifying low risk patients for whom further cardiovascular testing should be deferred.

## Methods

### Study cohort

We have previously published details of the study cohort [4, 17]. In brief, after excluded patients with acute coronary syndrome, previous CAD, insufficient image quality, missing baseline data, non-sinus rhythm, structural heart disease, heart failure and > 90 years old, 5289 consecutive patients referred to CCTA for SCP suspected of obstructive CAD were included in the final analysis in a regional cardiovascular center recognized as tertiary A level (Tianjin Chest Hospital, Tianjin, China) from December 2015 to December 2017. All patients were followed up until December 2019. This observational study complied with the Declaration of Helsinki and was approved by Tianjin Chest Hospital Ethics Committee.

### Definitions of baseline characteristics

Baseline clinical data including age, sex, diabetes mellitus, hypertension, hyperlipidemia, smoking, family history of premature CAD and type of SCP were prospectively collected and defined as described previously [4, 17].

### PTP models and risk assessment strategies

The PTP of obstructive CAD were estimated using ESC-PTP model (age, sex and type of SCP) [9] and CACS-CL model (age, sex, type of SCP, diabetes mellitus, hypertension, hyperlipidemia, family history and CACS) [14] as previously reported, respectively.

**Fig. 1:**
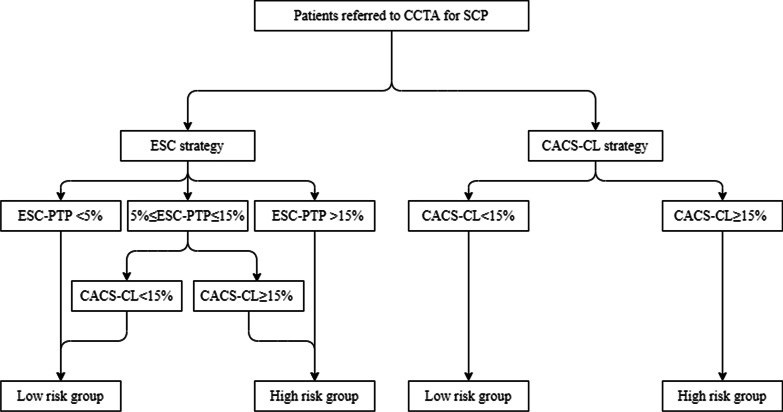
Flow chart. *ESC* European Society of Cardiology, *PTP* pretest probability, *ESC strategy* 2019 European Society of Cardiology guideline-determined risk assessment strategy, *CACS-CL* coronary artery calcium score-weighted clinical likelihood, *CACS-CL strategy* coronary artery calcium score-weighted clinical likelihood model-based risk assessment strategy, *CCTA* coronary computed tomographic angiography

According to current guidelines, cardiovascular testing should be deferred for a low risk patient and the impact of estimation of PTP to outcome was tested by splitting patients to different risk groups according to ESC-PTP or CACS-CL model. Details of risk groups according to ESC and CACS-CL strategy were illustrated in Fig. 1 and as follows:

ESC strategy: For patients with ESC-PTP between 5 and 15%, we selected CACS-CL model [14]. Thus, patients with ESC-PTP < 5% or 5% ≤ ESC-PTP ≤ 15% and CACS-CL < 15% were divided into low risk group and the other patients were classified into high risk group. CACS-CL strategy: we divided patients with CACS-CL ≥ 15% into high risk group and the other patients into low risk group.

### CACS and CCTA

Procedure details of CACS and CCTA have been previously described [4, 17]. A noncontrast CACS scan was acquired to quantify CACS before every CCTA scan and CACS was determined by two blinded observers, a radiologist and a cardiologist using Agatston method [18]. The major parameters for CCTA have been previously described [4, 17, 19]: detector collimation of 2 × 128 × 0.6 mm, slice thickness of 0.6 mm, gantry rotation time of 280 ms, heart rate adaptive pitch of 0.2–0.5, tube current of 290–560 mAs/rotation and tube voltage of 80–120 kV. Three blinded observers, two radiologist and a cardiologist, evaluated the CCTA data. All segments ≥ 2 mm in diameter were analyzed and the maximal degree of coronary diameter stenosis was defined as 0%, 1–49% and ≥ 50%. Nonobstructive CAD was defined as present if a patient had at least one lesion with 1–49% diameter stenosis and no lesion with ≥ 50% diameter stenosis. Obstructive CAD was defined as present if a patient had at least one lesion with ≥ 50% diameter stenosis or any unassessable segments because of severe calcification on CCTA. All interobserver disagreements were resolved by consensus.

### Follow up and endpoints

After CCTA, the local investigators made the subsequent clinical decisions for clinical management, such as further test including invasive coronary angiography ICA and clinical interventions like optimal medication treatment and coronary revascularization, based on recommendations from Coronary Artery Disease–Reporting and Data System [20], other clinical guidelines [9, 21] and the local institutional protocols [4, 17]. Contact information of all patients including telephone number, E-mail address and home address were collected before CCTA. All patients were followed up until December 2019 and follow-up information was obtained by phone call or physician interview at 6, 12, 24, 36 and 48 months after CCTA. Major adverse cardiovascular event (MACE) was defined as a composite of cardiac death and nonfatal myocardial infarction. Cardiac death was defined as any death caused by cardiac disease or for which no other cause could be found. Myocardial infarction was defined according to the Fourth Universal Definition of Myocardial Infarction [22]. The utilization of invasive procedures within 60 days after CCTA, included ICA and coronary revascularization (CR) were identified on electronic medical system. All endpoints were adjudicated via review of follow-up information and medical records by an independent clinical event committee who were blinded to other data.

### Statistical analysis

All statistical analyses were performed by MedCalc (version 15.2.2) and R (version 3.2.4). Two-tailed *p* < 0.05 was considered statistically significant. Differences for continuous data were compared using Student’s t-test or Mann Whitney U-test as appropriate. Categorical variables were compared using χ^2^ test or Fisher exact test as appropriate.

We used discrimination and calibration to validate and compare ESC-PTP model and CACS-CL model [23]. Discrimination was the degree to which a model separates between positive and negative patients and manifested by the area under receiver-operator characteristic curve (AUC) and integrated discrimination improvement (IDI) [24]. Calibration was analyzed by Hosmer–Lemeshow tests which divided patients into ten groups according to deciles of PTP and calculated a chi-square statistic (H–L χ^2^) to determine how well model fit the actual prevalence [23].

Net reclassification improvement (NRI) was used to determine how a risk assessment strategy reclassified patients into various risk groups compared with another [24]. Kaplan–Meier curves were generated for cumulative event-free estimates survival from MACE and were compared by log-rank test. Cox proportional hazards regression models were used to calculate hazard ratios (HRs) and 95% confidence intervals (CIs), which assessed association of risk groups to the time to the first MACE (or censoring). To further investigate the impact of CACS on downstream clinical management, we used the ratio for No. of additional CACS scans/No. of patients reclassified into low risk group when applying CACS-CL strategy instead of ESC strategy.

## Results

### Baseline characteristics grouped by CCTA result

Table 1 shows baseline characteristics of study cohort according to the presence of obstructive CAD. The cohort consisted of 5289 patients and the mean age was 56.1 years. Of these patients, 51% (2697/5289) were males and 19% (1005/5289) had obstructive CAD. Among patients with a CACS of 0, 103 had had obstructive CAD. All variables were positively associated to the presence of obstructive CAD.

**Table 1:** Baseline characteristics grouped by CCTA result

Characteristic	Total	Obstructive CAD^a^		*p*
	n = 5289	Yes (n = 1005)	No (n = 4284)	
Age (years, mean ± SD)	56.1 ± 10.3	59.3 ± 10.5	55.4 ± 10.3	< 0.0001
Male sex	2697 (51)	603 (60)	2094 (49)	< 0.0001
Diabetes	1005 (19)	241 (24)	764 (18)	< 0.0001
Hypertension	2169 (41)	462 (46)	1707 (40)	0.0004
Hyperlipidemia	1798 (34)	392 (39)	1406 (33)	0.0002
Smoking	1534 (29)	332 (33)	1202 (28)	0.0018
Family History	1904 (36)	422 (42)	1482 (36)	< 0.0001
Symptom				< 0.0001
Nonanginal chest pain	2010 (38)	161 (16)	1849 (43)	
Atypical anginal	2380 (45)	482 (48)	1898 (44)	
Typical anginal	899 (17)	362 (36)	537 (13)	
CACS (median, 25th–75th)	4 (0–75)	50 (0–362)	0 (0–44)	< 0.0001
0	2486 (47)	103 (10)	2383 (56)	< 0.0001
> 0	2803 (53)	902 (90)	1901 (44)	< 0.0001

Values are presented as n (%) unless stated otherwise

*SD* standard deviation, *CAD* coronary artery disease; CCTA: coronary computed tomographic angiography, *CACS* coronary artery calcium score

^a^Obstructive CAD was defined as an individual had at least one lesion with ≥ 50% diameter stenosis or any non-assessable segments because of severe calcification on CCTA

### Comparison of ESC-PTP and CACS-CL model

Comparison of discrimination for ESC-PTP and CACS-CL model using AUC and IDI is presented in Table 2. The AUC for CACS-CL model was significantly larger than that for ESP-PTP model (0.838 versus 0.735, *p* < 0.0001). Compared to ESP-PTP model, CACS-CL model demonstrated a positive IDI (9%, < 0.0001).

**Table 2:** Discriminations of CACS-CL and ESC-PTP model

	AUC			IDI			
	Statistic	95% CI	*p*	PTP		Statistic^b^	*p*
				Positive^a^ (%)	Negative (%)		
ESC-PTP model	0.735	0.722 to 0.748	< 0.0001	42%	19%	9%	< 0.0001
CACS-CL model	0.838	0.827 to 0.848		45%	13%		

*AUC* Area under the receiver operating characteristic curve, *IDI* integrated discrimination improvement, *CI* confidence interval, *CCTA* coronary computed tomographic angiography, *CACS-CL* coronary artery calcium score–weighted clinical likelihood, *PTP* pretest probability, *ESC* 2019 European Society of Cardiology guideline

^a^Positive patient was defined as an individual had at least one lesion with ≥ 50% diameter stenosis or any non-assessable segments because of severe calcification on CCTA

^b^Compared to ESC-PTP model, the IDI of CACS-CL model = [P(CACS-CL|Positive)−P(ESC-PTP|Positive)]−[P(CACS-CL|Negative)−P(ESC-PTP|Negative)]

**Fig. 2:**
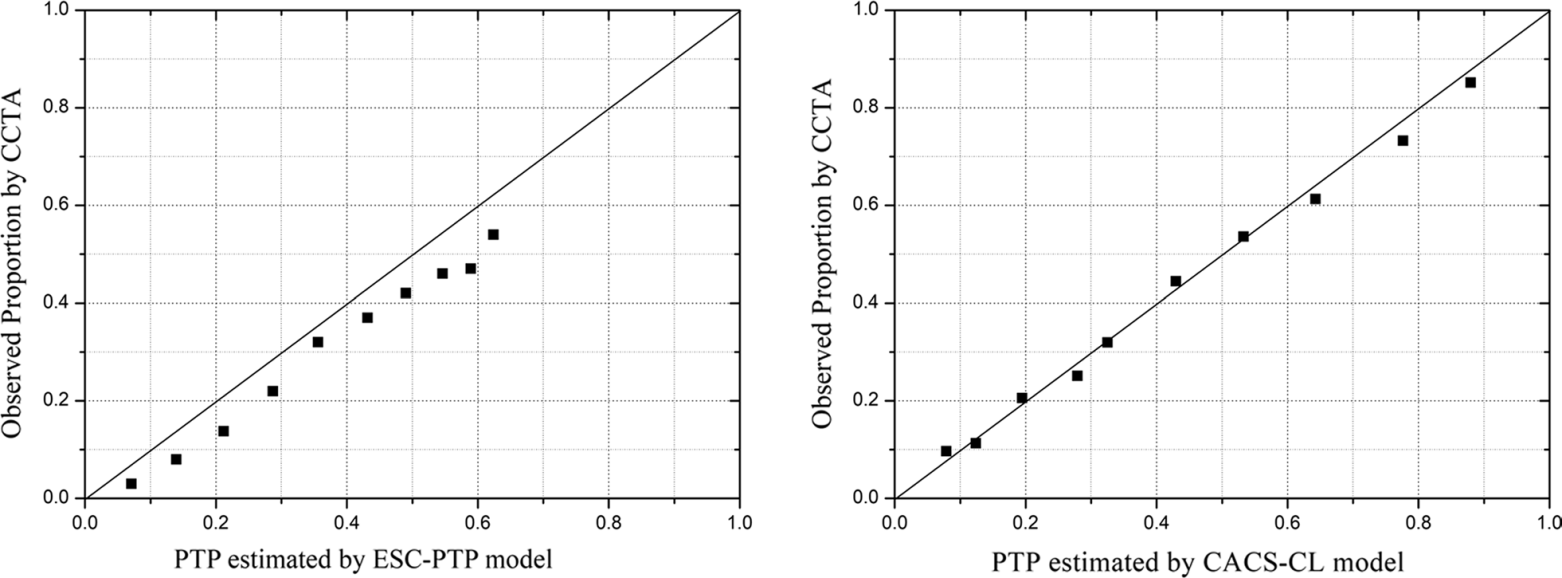
Model-specific PTP and observed probability by deciles of PTP. *CAD* coronary artery disease, *ESC* European Society of Cardiology, *PTP* pretest probability, *ESC strategy* 2019 European Society of Cardiology guideline-determined risk assessment strategy, *CACS-CL* coronary artery calcium score-weighted clinical likelihood, *CACS-CL strategy* coronary artery calcium score-weighted clinical likelihood model-based risk assessment strategy, *CCTA* coronary computed tomographic angiography

Figure 2 illustrated the comparison of predicted and observed probabilities of obstructive CAD by deciles of PTP. Graphically, ESC-PTP model overestimated the probability of obstructive CAD, with predicted values higher than those observed. As a result, calibration of ESC-PTP model was poor (H–L χ^2^ = 95.46, *p* < 0.0001), but CACS-CL model demonstrated less discordance (H–L χ^2^ = 28.74, *p* < 0.0001).

### Comparison of ESC and CACS-CL strategy

According to CACS-CL strategy, 58% (3042/5289) were assigned to low risk group. Among 1479 patients with an ESC-PTP between 5 and 15%, 813 patients had a CACS-CL < 15%. Together with the 1514 patients with an ESC-PTP below 5%, ESC strategy totally classified 44% (2327/5289) into low risk group.

Table 3 shows the distribution of baseline characteristics, CAD and MACE by low and high risk group based on two strategies. In terms of both ESC and CACS-CL strategy, there were statistically significant differences for all baseline characteristics. In low and high risk group based on ESC strategy, 34% (801/2327) and 62% (1844/2962) patients had evidence of some degree of CAD on CCTA, with 7% (153/2327) and 29% (852/2962) patients having obstructive CAD, respectively. The percentages were 31% (957/3042), 75% (1688/2247), 4% (134/3042) and 39% (871/2247) for CACS-CL strategy, respectively. The distribution of CAD according to risk groups was significantly different (*p* < 0.0001 for two strategies, respectively). During the follow-up of 26 (interquartile range: 13 to 38) months, 211 patients experienced MACE: 24 patients died from cardiac cause and 187 patients suffered from nonfatal MI. Compared with patients in low risk group, patients in high risk group were more likely to have MACE (ESC strategy: 6% versus 1%, *p* < 0.0001 and CACS-CL strategy: 8% versus 1%, *p* < 0.0001).

**Table 3:** Baseline characteristics, CAD and MACE by risk groups based on two strategies

	Total	ESC strategy		*p*	CACS-CL strategy		*p*
		High risk	Low risk		High risk	Low risk	
	n = 5289	n = 2962	n = 2327		n = 2247	n = 3042	
Age (years, mean ± SD)	56.1 ± 10.3	58.1 ± 10.4	53.6 ± 10.6	< 0.0001	58.6 ± 10.5	54.3 ± 10.4	< 0.0001
Male	2697 (51)	1807 (61)	890 (38)	< 0.0001	1303 (58)	1394 (46)	< 0.0001
Diabetes	1005 (19)	652 (22)	353 (15)	< 0.0001	562 (25)	443 (14)	< 0.0001
Hypertension	2169 (41)	1303 (44)	866 (37)	< 0.0001	1011 (45)	1158 (38)	< 0.0001
Hyperlipidemia	1798 (34)	1155 (39)	643 (28)	< 0.0001	854 (38)	944 (31)	< 0.0001
Smoking	1534 (29)	948 (32)	586 (25)	< 0.0001	742 (33)	792 (26)	< 0.0001
Family history	1904 (36)	1214 (41)	690 (30)	< 0.0001	989 (44)	915 (30)	< 0.0001
Symptom				< 0.0001			< 0.0001
Nonanginal chest pain	2010 (38)	918 (31)	1092 (47)		742 (33)	1268 (42)	
Atypical angina	2380 (45)	1392 (47)	988 (42)		989 (44)	1391 (46)	
Typical angina	899 (17)	652 (22)	247 (11)		516 (23)	383 (12)	
CACS (median, 25th–75th)	4 (0–75)	55 (0–389)	0 (0–37)	< 0.0001	62 (0–401)	0 (0–31)	< 0.0001
CAD				< 0.0001			< 0.0001
No CAD	2644 (50)	1118 (38)	1526 (65)		559 (25)	2085 (69)	
Nonobstructive CAD	1640 (31)	992 (33)	648 (28)		817 (36)	823 (27)	
Obstructive CAD^a^	1005 (19)	852 (29)	153 (7)		871 (39)	134 (4)	
MACE	211 (4)	177 (6)	34 (1)	< 0.0001	185 (8)	26 (1)	< 0.0001

Values are presented as n (%) unless stated otherwise

*ESC strategy* 2019 European Society of Cardiology guideline-determined risk assessment strategy, *CACS* coronary artery calcium score, *CACS-CL strategy* CACS-weighted clinical likelihood model-based risk assessment strategy, *CAD* coronary artery disease, *MACE* major adverse cardiovascular event

^a^Obstructive CAD was defined as an individual had at least one lesion with ≥ 50% diameter stenosis or any non-assessable segments because of severe calcification on CCTA

**Fig. 3:**
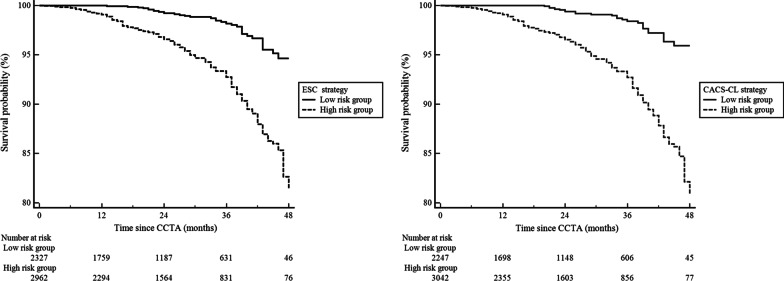
Kaplan–Meier curves comparing high and low risk groups determined by four strategies. *ESC* European Society of Cardiology, *PTP* pretest probability, *ESC strategy* 2019 European Society of Cardiology guideline-determined risk assessment strategy, *CACS-CL* coronary artery calcium score-weighted clinical likelihood, *CACS-CL strategy* coronary artery calcium score-weighted clinical likelihood model-based risk assessment strategy, *CCTA* coronary computed tomographic angiography

Figure 3 exhibitions Kaplan–Meier curves comparing survival probabilities in high and low risk groups. High risk group according to ESC strategy and CACS-CL strategy demonstrated a significantly higher risk of MACE, respectively (log-rank test: *p* < 0.0001 for ESC strategy and *p* < 0.0001 for CACS-CL strategy). The association of risk groups determined by CACS-CL strategy (high versus low) with MACE was stronger (HR for ESC strategy: 3.97, 95% CI: 2.75 to 5.72, *p* < 0.0001 and HR for CACS-CL strategy: 5.11, 95% CI: 3.40 to 7.69, *p* < 0.0001).

Table 4 is the reclassification table to compare ESC strategy and CACS-CL strategy. Among the 4284 negative patients, compared to ESC strategy, CACS-CL strategy reclassified 801 from high to low risk group, but 67 from low to high. Of the 1005 positive patients, 32 were reclassified to high risk group but 13 to low. As a result, the NRI comparing CACS-CL strategy to ESC strategy was 17% in negative, 2% in positive, and 19% in all (*p* < 0.0001).

**Table 4:** Reclassification table comparing CACS-CL to ESC strategy

	Risk groups by CACS-CL strategy		Total	Reclassification^a^		NRI^b^	*p*
	Low	High		Up	Down		
Risk groups by ESC strategy							
Negative patients				2%	19%	19%	< 0.0001
Low	2107	67	2174				
High	801	1309	2110				
Total	2908	1376	4284				
Positive patients^c^				3%	1%		
Low	121	32	153				
High	13	839	852				
Total	134	871	1005				

*CACS-CL strategy* CACS-weighted clinical likelihood model-based risk assessment strategy, *ESC strategy* 2019 European Society of Cardiology guideline-determined risk assessment strategy, *CCTA* coronary computed tomographic angiography, *CAD* coronary artery disease, *NRI* net reclassification improvement

^a^The classification of patients by CACS-CL strategy was compared to that by CACS strategy

^b^NRI = [P(Up|Positive)−P(Down|Positive)]−[P(Up|Negative)−P(Down|Negative)]

^c^Positive patient was defined as an individual had at least one lesion with ≥ 50% diameter stenosis or any non-assessable segments because of severe calcification on CCTA

In total, 1143 patients had ICA based on CCTA and 328 patients underwent CR. Compared with low risk patients, high risk patients had more ICA (ESC strategy: 31% (924/2962) versus 9% (219/2327), *p* < 0.0001; CACS-CL strategy: 39% (882/2247) versus 9% (261/3042), *p* < 0.0001) and CR (ESC strategy: 8% (249/2962) versus 3% (79/2327), *p* < 0.0001; CACS-CL strategy: 11% (244/2247) versus 3% (84/3042), *p* < 0.0001).

### The impact of CACS on ESC and CACS-CL strategy

ESC strategy classified 49% (2110/4284) negative patients into high risk group, for which further cardiovascular testing were recommend according to current guidelines. The application of CACS-CL strategy instead of ESC strategy would result in a prominently change of diagnostic strategy: 38% (801/2110) of these patients were reclassified into low risk group, for which no further cardiovascular testing was recommend. Moreover, during follow-up, only 2 nonfatal MI occurred among the 801 patients. Based on ESC strategy, 28% of the 5289 patients with borderline ESC-PTP would require further CACS scan, with an additional (5289–1479 = 3810) CACS scans required if following the CACS-CL strategy. In a word, the replacement of ESC strategy by CACS-CL strategy would avoid 38% unnecessary cardiovascular testing and avoid an unnecessary cardiovascular testing at the expense of 3810/801≈5 additional CACS scans.

## Discussion

In this CCTA-based analysis of patients with SCP suggestive of CAD, compared to ESC-PTP model, CACS-CL model revealed a larger AUC, a positive IDI and less discrepancy between observed and predicted probabilities. Both ESC strategy and CACS-CL strategy classified a proportion of patients into low risk groups with low prevalence of CAD and MACE, but the additional use of CACS in CACS-CL strategy improved the identification of patients who may derived minimal benefit from further cardiovascular testing.

Although the concept of PTP has been recognized as the cornerstone of SCP clinical management for decades [3, 25], numerous external validation studies suggested that the traditional invasive ICA-based approaches to PTP revealed significant overestimation of the actual prevalence of CAD [4–6, 26], which accounted, in part, for the substantial number of unnecessary cardiovascular testing [27–29]. To improve the estimation of PTP, Knuuti et al. developed the ESC-PTP model [1] based on data from 3 contemporary cohorts with a total of 22 366 SCP patients referred for CCTA [10, 11, 26] and the diagnostic performance of ESC-PTP model has been validated externally [12–15]. Moreover, ESC guideline suggested a novel strategy incorporating CACS with estimation of PTP, especially for patients with borderline ESC-PTP [9].

Although the diagnostic and prognostic value of CACS have been established [30, 31], extensive literature has repeatedly demonstrated the strong interplay between CACS and risk factors for predicting the presence of obstructive CAD and for future MACE [17, 32–34]. In conformity with previous findings comparing CACS-based PTP models to other models [4, 6], we demonstrated that CACS-CL model offered precise estimation of PTP and prediction of MACE. As far as we know, this is the first comparative description of two different strategies which both included CACS-CL model: in contrast to ESC strategy, CACS-CL strategy applied CACS scan to all SCP patients. A recent study with a shorter follow-up selected CAD Consortium extended model (CCEM), which also incorporated clinical variables plus CACS, for further risk assessment in patients with borderline ESC-PTP [35]. However, the CCEM was a traditional approach to estimate pretest probability (PTP) based on ICA [36] and has been demonstrated to obviously overestimate the actual prevalence of CAD according to the calibration plots [14].

In the present study, compared to ESC strategy, CACS-CL strategy seemed to be associated with greater effectiveness in identifying patients at low risk for whom further cardiovascular testing should be deferred, resulting from the superiority for the diagnosis of CAD and prediction of MACE. More importantly, the replacement of ESC strategy by CACS-CL strategy would avoid an unnecessary cardiovascular testing at the expense of (5289–1479)/801 = 5 additional CACS scans. Greater emphasis should be placed on this management decision paradigm incorporating CACS-based estimation of PTP to all SCP patients, because of low radiation exposure and scan costs, no need for provocation, vessel puncture or contrast, few contraindications, and little difficulty in operation and interpretation of CACS scan [37]. It also bears mentioning that comparison of cost-effectiveness between this attractive risk assessment strategy and sequential instrument incorporating CACS-based PTP estimation only to subgroups of SCP patients is needed in the future.

## Limitations of the study

Several limitations needed attention. First, the present study was subjected to the observational design. The clinical decision before and after CCTA were made by local physicians and the substantial selection biases should not be ignored, such as a significant number of low risk patients not being included, as well as local referral pattern bias of CTA versus functional testing or ICA. Thus, the generalizability of the present findings in patients who did not undergo CCTA need further study. Second, the ESC guideline also recommended other new predictors, such as changes on rest or exercise electrocardiogram (ECG) and LV dysfunction exercise to improve estimation of PTP. However, CACS provided the most incremental diagnosis and prognosis information above traditional cardiovascular risk factors [6, 36]. Third, we defined unassessable segments as positive ones because further testing should usually be referred for nonconclusive CCTA. Fourth, this analysis focused on the presence of coronary diameter stenosis ≥ 50%. Evaluation of high risk CAD, such as left main disease or 3-vessel disease with maximal degree of coronary diameter stenosis ≥ 70% would be helpful to identify patients who may derive maximal benefit from CR [38–40]. However, our data also supported the potential of CACS-CL strategy to optimize the downstream utilization of invasive procedures. Fifth, although both the ESC [1] and CACS-CL [14] strategy included patients presenting with dyspnea, this study only focused on SCP. Thus, the conclusions should not be extrapolated to patients with dyspnea or acute chest pain, or asymptomatic patients.

## Conclusions

In conclusion, CACS-CL model provided precise estimation of PTP, resulting in the superiority of CACS-CL strategy in identifying patients at low risk who may derive minimal benefit from further cardiovascular testing. Moreover, compared to ESC strategy, CACS-CL strategy might have more potential to defer unnecessary cardiovascular testing at a low expense. For risk assessment of patients presenting with SCP suggestive of obstructive CAD, the cost-effectiveness of CACS-CL strategy which obviously increase the use of CACS scan needs to be comprehensive validated and compared with other strategies in the future.

## Data Availability

The datasets generated and/or analyzed during the current study are not publicly available due to the ongoing project but are available from the corresponding author on reasonable request.

## Abbreviations

SCP: Stable chest pain
PTP: Pretest probability
ICA: Invasive coronary angiography
CAD: Coronary artery disease
CACS: Coronary artery calcium score
CCTA: Coronary computed tomography angiography
CACS-CL: CACS-weighted clinical likelihood
CACS-CL strategy: CACS-CL model alone-based risk assessment strategy
ESC strategy: 2019 ESC guideline-determined risk assessment strategy
AUC: Area under receiver-operator characteristic curve
IDI: Integrated discrimination improvement
H–L χ^2^: Hosmer–Lemeshow chi-square statistic
NRI: Net reclassification improvement
HR: Hazard ratio
CI: Confidence intervals
MACE: Major adverse cardiovascular event
CCEM: CAD Consortium extended model
